# Using the *Daphnia magna* Transcriptome to Distinguish Water Source: Wetland and Stormwater Case Studies

**DOI:** 10.1002/etc.5392

**Published:** 2022-08-09

**Authors:** Mark D. Jankowski, David J. Fairbairn, Joshua A. Baller, Benjamin M. Westerhoff, Heiko L. Schoenfuss

**Affiliations:** ^1^ Minnesota Pollution Control Agency St. Paul Minnesota USA; ^2^ Veterinary Population Medicine Department University of Minnesota—Twin Cities St. Paul Minnesota USA; ^3^ US Environmental Protection Agency Seattle Washington USA; ^4^ Minnesota Supercomputing Institute University of Minnesota—Twin Cities Minneapolis Minnesota USA; ^5^ Aquatic Toxicology Laboratory St. Cloud State University St. Cloud Minnesota USA

**Keywords:** Biomonitoring, Ecotoxicogenomics, Effects‐based monitoring, Invertebrate toxicology, Transcriptomics

## Abstract

A major challenge in ecotoxicology is accurately and sufficiently measuring chemical exposures and biological effects given the presence of complex and dynamic contaminant mixtures in surface waters. It is impractical to quantify all chemicals in such matrices over space and time, and even if it were practical, concomitant biological effects would not be elucidated. Our study examined the performance of the *Daphnia magna* transcriptome to detect distinct responses across three water sources in Minnesota: laboratory (well) waters, wetland waters, and storm waters. Pyriproxyfen was included as a gene expression and male neonate production positive control to examine whether gene expression resulting from exposure to this well‐studied juvenoid hormone analog can be detected in complex matrices. Laboratory‐reared (<24 h) *D. magna* were exposed to a water source and/or pyriproxyfen for 16 days to monitor phenotypic changes or 96 h to examine gene expression responses using Illumina HiSeq 2500 (10 million reads per library, 50‐bp paired end [2 × 50]). The results indicated that a unique gene expression profile was produced for each water source. At 119 ng/L pyriproxyfen (~25% effect concentration) for male neonate production, as expected, the Doublesex1 gene was up‐regulated. In descending order, gene expression patterns were most discernable with respect to pyriproxyfen exposure status, season of stormwater sample collection, and wetland quality, as indicated by the index of biological integrity. However, the biological implications of the affected genes were not broadly clear given limited genome resources for invertebrates. Our study provides support for the utility of short‐term whole‐organism transcriptomic testing in *D. magna* to discern sample type, but highlights the need for further work on invertebrate genomics. *Environ Toxicol Chem* 2022;41:2107–2123. © 2022 The Authors. *Environmental Toxicology and Chemistry* published by Wiley Periodicals LLC on behalf of SETAC.

## INTRODUCTION

A major challenge in ecotoxicology is accurately and sufficiently measuring chemical exposure and biological effect given the presence of complex and dynamic contaminant mixtures in surface waters and storm water runoff (Fairbairn et al., 2018; Riva et al., 2019; Tousova et al., 2017). It is impractical to quantify all chemicals in such matrices over space and time, and even if it were practical, biological effects would not be known. Instead, whole‐organism (Mehinto et al., 2021) or in vitro (Blackwell et al., 2018) bioassays can be deployed to ascertain the biological effects of environmental mixtures. Whole‐organism assays, like whole‐effluent toxicity (WET) testing (US Environmental Protection Agency [USEPA], 2000), assess the effects of mixtures on organism‐level outcomes (e.g., survival, growth, and reproduction) but do not provide mechanistic insights such as the potential for endocrine disruption (Chapman, 2000; Escher et al., 2018). Furthermore, vertebrate‐based whole‐organism tests increasingly elicit ethical concerns, in part because of the large number of animals used for routine testing. New tools are therefore needed to simultaneously measure organismal and mechanistic effects of exposure to complex chemical mixtures inherent to most source waters.

Comprehensive understanding of effects in organisms exposed to environmental contaminants requires models that represent the relevant biological domains and subdomains, and capture genomic, transcriptional, transgenerational, and other sublethal effects (Brun et al., 2019). Invertebrates are potential contaminant receptors and integral to aquatic communities. However, many bioeffects‐based monitoring tools are vertebrate‐focused (Blackwell et al., 2018; Eads et al., 2007). Until recently, molecular studies and tools focusing on invertebrates (e.g., *Daphnia*, *Hyalella*, *Hydra*, *Lymnaea*, *Potamopyrgus*) have received less attention and development (Segner et al., 2003), especially for endocrine‐related pathways and effects in noninsect arthropods (e.g., *Daphnia*; Miyakawa et al., 2018). As the routine use of vertebrates in toxicological testing is deemphasized (National Research Council, 2007), there is a need for alternative methods, including the use of short‐duration invertebrate tests.

Conventional water quality studies have used *Daphnia magna*–based methods and models extensively for decades (Brun et al., 2019). *Daphnia magna* are amenable to laboratory culturing and cloning and act as aquatic ecosystem sentinels relevant to a broad range of geographic and in situ conditions (Miyakawa et al., 2018). *Daphnia* spp. were among the first aquatic indicator species with a fully sequenced genome (Colbourne et al., 2011; Lee et al., 2019), an essential resource for a robust interpretation of genomic data. Transcriptomics by way of RNA sequencing (RNAseq) is a comprehensive, unbiased, rapid, and cost‐effective tool to detect genes and biological pathways responsive to chemicals and environmental mixtures in many organisms. If this type of effects‐based monitoring can be adapted to invertebrate species, it will provide a valuable tool to water resource managers to assess potential adverse biological effects in surface waters without the need for extensive water chemical analysis or use of vertebrate exposure studies. Although RNAseq has been used across a wide array of environmental venues (Altenburger et al., 2015; Schroeder et al., 2016), its applications in invertebrate toxicology and environmental analysis are less developed. Our study addresses this information gap by examining the performance of an invertebrate‐based transcriptomic approach in the context of complex yet commonly occurring environmental mixtures in urban wetlands and storm water.

Wetlands, especially in urban settings, are important to biodiversity and support a variety of organisms, including sensitive and endangered species (Hook, 1993). However, natural, restored, and constructed wetlands frequently receive sanitary or industrial wastewater and/or stormwater runoff (Hook, 1993; Matamoros et al., 2012). Like lakes, wetlands typically have longer hydraulic retention times than fluvial systems, and thus accumulate contaminants (Matamoros et al., 2012). As such, wetlands often contain complex and variable chemical mixtures (e.g., pesticides, nutrients, wastewater indicators, and metals; Zhang et al., 2014); this situation complicates our understanding of the biological effects and environmental risks most likely to impact organisms at a given wetland. Thus, an evaluation of bioassays capable of assessing an expansive domain of biological processes is desired. Urban wetlands may also be treated with insecticide applications including pyriproxyfen for larval mosquito control (Centers for Disease Control and Prevention [CDC], 2020; Hustedt et al., 2020; Vieira Santos et al., 2017). The mode of action of pyriproxyfen appears to involve interaction with the crustacean methyl farnesoate receptor (MfR), which results in juvenile hormone–like behavior (LeBlanc et al., 2013; Olmstead & LeBlanc, 2003) and ultimately affects daphnid reproduction. By spiking laboratory and wetland water with pyriproxyfen, we examined the hypothesis that genes associated with the MfR pathway such as doublesex1 (*dsx1*; Kato et al., 2011; Song & Tollefsen, 2021) would be differentially expressed in both simple and complex chemical mixtures. If supported, our study would demonstrate that known bioactivities of a positive control compound could be detected with short‐term exposures of daphnids using RNAseq, thus enhancing our confidence that the approach is sufficiently robust to apply to complex environmental mixtures.

Complex and dynamic chemical mixtures are also present in urban storm waters (Fairbairn et al., 2018; Masoner et al., 2019; Peter et al., 2018). Typically, most urban‐area precipitation is not intercepted or infiltrated on‐site but flows over impervious surfaces as runoff (stormwater) to engineered structures (e.g., sewers, stormwater ponds) or directly to receiving surface waters. Because runoff may mobilize and transport chemicals from anything it contacts, myriad terrestrial and near‐surface materials (e.g., buildings/infrastructure, vehicles/exhaust, roads, parking lots, lawns, athletic fields, litter, and exposed commercial/industrial storage areas, tanks, and spills) may be contaminant sources to stormwater. For example, a common tire compound, *N*‐(1,3‐dimethylbutyl)‐*N*′‐phenyl‐*p*‐phenylenediamine ozonated to 2‐anilino‐5‐[(4‐methylpentan‐2‐yl)amino]cyclohexa‐2,5‐diene‐1,4‐dione, was recently identified as lethal to coho salmon (*Oncorhynchus kisutch*) and has been detected in urban storm waters of the Pacific Northwest (Tian et al., 2021) and beyond (Rauert et al., 2022; Seiwert et al., 2022). The few published studies of contaminants of emerging concern (CECs) in stormwater have reported complex contaminant mixtures with chemicals and concentrations typical of treated municipal wastewater (e.g., nonprescription pharmaceuticals, personal care products, urban‐ or residential‐use pesticides, and manufacturing‐industrial compounds (e.g., flame retardants, benzotriazoles; Fairbairn et al., 2018; Wicke et al., 2016). Yet, like wetlands, surprisingly few stormwater–based bioeffect studies exist (Fairbairn et al., 2018; Mehinto et al., 2021; Westerhoff et al., 2018). The few stormwater–based studies of sublethal toxicity have reported estrogenic, molecular, transcriptional, and/or organismal effects comparable to those present in organisms exposed to treated wastewater (Bai et al., 2018; Bertucci et al., 2018; Martinovic‐Weigelt et al., 2018; McIntyre et al., 2016; Schultz et al., 2013; Seward, 2014). However, recent demonstrations of reduced mortality in coho salmon exposed to biofiltration‐treated versus untreated stormwater (Spromberg et al., 2016) indicate the value of using bioassays to examine the efficacy of treatment technologies.

**Figure 1:**
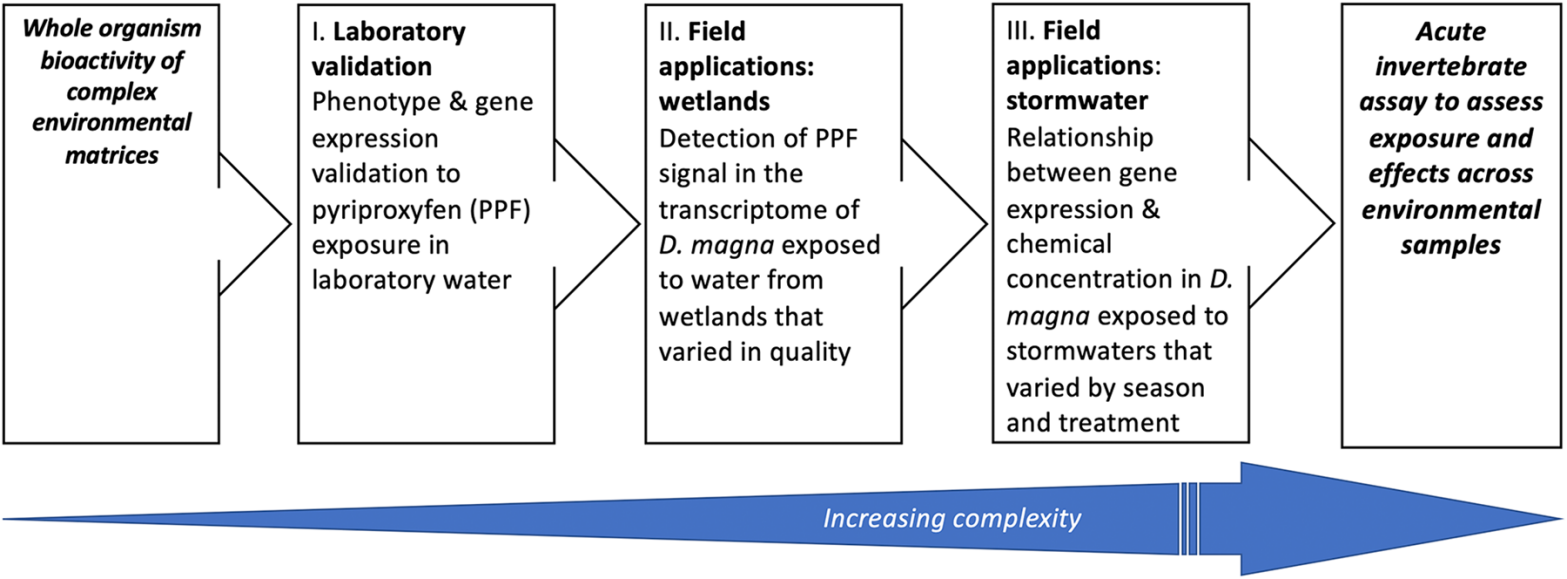
Conceptual diagram showing the three experimental steps of increasing complexity that were used to investigate the performance of an acute *Daphnia magna* transcriptomics assay for water quality.

Therefore, the objectives of the present study were to examine the utility of an acute invertebrate assay using three experimental steps of increasing complexity (Figure 1) and to ascertain whether “biological fingerprints” of chemical exposure can be detected and linked to apical biological effects in an invertebrate, *D. magna*. Our analysis focuses on transcriptomic and phenotypic effects in *D. magna* exposed to a known endocrine disruptor (pyriproxyfen), wetland waters, or untreated/treated stormwater samples. We draw linkages between exposure chemicals, differentially expressed genes (DEGs), and reproductive outcomes. This information can help elucidate the nature of the biological processes in *Daphnia* affected by exposure to these two important and chemically complex environmental matrices. Further, our study demonstrates the relative benefits and challenges of employing invertebrate transcriptomics compared to extensive chemical analysis in environmental monitoring.

## METHODS

### Experimental design

*Daphnia magna* was selected as the model organism for the present study because it is regularly used in environmental toxicity testing and environmental monitoring programs. In addition, the genome (Lee et al., 2019) and transcriptome (Orsini et al., 2016) of *D. magna* were recently sequenced and partially annotated. Our experimental study involved three components (Figure 1): 1) laboratory validation, a laboratory study to develop a low‐effects concentration of pyriproxyfen for male neonate production in laboratory‐reared *D. magna* for use as a reference compound for transcriptomic effects in subsequent wetland studies; 2) field applications (wetlands), exposure of laboratory‐reared *D. magna* to samples collected from wetlands with different indices of biological integrity (IBIs) based on macroinvertebrate and plant communities (Genet & Bourdaghs, 2006); and 3) field applications (stormwater), exposure of laboratory‐reared *D. magna* to water samples collected from stormwater management infrastructural locations that differed by upstream/downstream within‐catchment position and presence/absence of iron‐enhanced sand filtration (IESF) stormwater control measure (SCM) treatment. Daphnid RNA for Components 2 and 3 was sequenced. The use of a reference compound (pyriproxyfen) was especially important in our studies because our exposure waters were from environmental sources that inevitably contained a mixture of bioactive chemicals. If the transcriptomic effects of pyriproxyfen were undetectable in the exposing mixtures, then the challenges of using RNAseq to detect the specific bioactivity of unknown mixtures as part of environmental monitoring programs, for example, in *Daphnia*, would be highlighted. In addition, assessing the transcriptomic response to uncharacterized mixtures in the presence of a compound with characterized effects (pyriproxyfen) may provide clues regarding the presence of other similarly acting chemicals in the mixture.

### Laboratory rearing of *D. magna*

Live *D. magna* (purchased from Carolina Biological Supply) were placed into aerated St. Cloud State University well water and maintained as an experimental culture under a 16:8‐h light:dark photoperiod. All test water conditions were within Organisation for Economic Co‐operation and Development (OECD) Test No. 211 (OECD, 2012) guidelines. *Daphnia magna* were fed yeast–cerophyll–trout chow produced in‐house using a modified version of USEPA Method 1002.0. Twenty grams of trout chow (Wards Science) were aerated in 2 L of ultrapure water for 7 days, and 20 g of cereal leaves (Carolina Biological Supply) in 2 L of ultrapure water were stirred for 24 h. Each preparation was passively settled for 30 min, then strained through a fine mesh filter, combined with 20 g of yeast, resuspended in 2 L of ultrapure water, and frozen (−20 °C).

### Experiment 1, laboratory validation: Phenotype and gene expression validation to pyriproxyfen exposure in laboratory water

#### Experimental procedures

An exposure concentration–response curve was generated for the effect of pyriproxyfen on male neonate production to determine a lowest‐observed‐effect concentration (LOEC) or a 25% effect concentration (EC25) for use in subsequent wetland experiments. Experimental procedures broadly adhered to OECD Test No. 211 (OECD, 2012) but were limited to a 16‐day exposure period. *Daphnia magna* neonates (<24 h old) were randomly distributed among seven treatment groups (0, 29, 59, 89, 119, 179, and 239 ng/L pyriproxyfen), with each treatment group consisting of forty 50‐ml beakers, each with 40 ml of treatment water and three neonates. Evaporated water was replenished daily to maintain nominal pyriproxyfen concentrations, and organisms were transferred to new treatment solutions every fourth day. On each treatment renewal day, pyriproxyfen (in 96% ethanol) was serially diluted. Each day during the 16‐day experiment, newly hatched neonates were sexed under 10× magnification based on the presence (males) or absence (females) of enlarged primary antennae (Ebert, 2005) and then discarded. Samples (1 L) from representative stock solutions made for each exposure concentration were analyzed for pyriproxyfen as described in the section *Chemical contaminants: Organic chemicals*. *Daphnia magna* survival rates for each treatment group were at least 94%. The LOEC and 10th, 20th, 25th, and 50th percentile effective concentrations associated with a statistically significant increase in male neonates (percentage) compared to controls were calculated. The LOEC was used in subsequent wetland experiments.

### Experiment 2, field applications, wetlands: Detection of a pyriproxyfen signal in the transcriptome of *D. magna* exposed to water from wetlands that varied in quality as quantified by an IBI

#### Wetland sampling design

In July 2015, six wetlands across the state of Minnesota were sampled based on their IBI score. Based on their IBI scores (Supporting Information, Table S1), three were high‐ and three were low‐quality wetlands. Samples (1 L per amber glass bottle) were collected 5–15 cm below the water surface from areas with minimal emergent vegetation, transported on ice in coolers, and stored frozen (−20 °C) in amber glass bottles. Water quality parameters were recorded at the time of sample collection (Supporting Information, Table S1).

#### Exposure of laboratory‐reared *D. magna* to wetland water samples

Water collected from the six wetlands was thawed for these experiments and filtered through Whatman #1 filters (11 µm; Sigma‐Aldrich) to remove coarse debris from the samples. Treatment groups (*n* = 15) included water from each wetland spiked with either 119 ng/L pyriproxyfen (in 96% ethanol) or ethanol vehicle control, laboratory water spiked with 119 or 239 ng/L pyriproxyfen, and a laboratory water‐only control group. For each treatment group, 10 replicate 50‐ml beakers, each containing three *D. magna* (<24 h old) and 40 ml of treatment water, were exposed for 96 h. Treatment‐specific water in each beaker was replenished daily to replace evaporated water. Laboratory water chemistry (dissolved oxygen, pH, specific conductivity, temperature) was measured daily to verify compliance with OECD 211 (OECD, 2012).

#### RNA extraction

At the end of the 96‐h exposure period, to obtain sufficient RNA quantity for sequencing, the 30 treated (less 0%–7% mortality) neonates were randomly divided into five groups of five individuals, then stored in 100 µl of RNAlater (catalog no. R0901; Sigma‐Aldrich) at −80 °C until RNA extraction. RNA was extracted by sonication (30 s), followed by extraction using the PureLink RNA Mini Kit (catalog no. 12183018 A; Thermo Fisher Scientific).

### Experiment 3, field applications, stormwater: Relationship between gene expression and chemical concentration in *D. magna* exposed to storm waters that varied by season and upgradient/downgradient within‐catchment position and presence/absence of IESF treatment

#### Stormwater sampling design

Nine sampled stormwater locations (Supporting Information, Table S2) comprised three representatives each of three different stormwater management infrastructural site types: *PIPE‐Untreated*, which represented untreated stormwater from relatively large catchments just above the various receiving waters (i.e., downstream within‐catchment position); *IESF‐Untreated*, which represented untreated stormwater influent to three IESFs that were relatively upgradient in their catchment; and *IESF‐Treated*, which represented the corresponding (paired) IESF‐treated effluents. Three seasonal runoff event “types” were sampled (*n* = 29 total stormwater samples): spring rainfall (April), early summer rainfall (May), and late summer rainfall (September). The actual sampling dates were selected based on antecedent and predicted weather and catchment conditions, anticipated contaminant profiles, and/or exposure periods of interest (e.g., early summer runoff would reflect urban and preemergent crop pesticide applications and near‐peak growth). Samples were collected in accordance with previously described chemical and biological methods (Fairbairn et al., 2018; Westerhoff et al., 2018). Water for *D. magna* exposures was collected in precleaned 1‐L polytetrafluoroethylene containers. Water quality parameters were recorded at the time of sample collection.

#### Exposure to stormwater samples

Exposure and RNA extraction procedures for stormwater and wetland samples were similar, except that no stormwater samples were spiked with pyriproxyfen.

#### Standard parameters of water chemistry

Wetland and/or stormwater samples were examined for basic water chemistry parameters in three ways. First, field measurements (pH, specific conductivity, temperature [°C], dissolved oxygen [milligrams per liter]) were taken with a YSI 556 MPS sonde. Second, wetland samples were evaluated by the Minnesota Department of Health Laboratory for suspended solids (SM2540, D‐1997), total alkalinity (SM2320, B‐1997), sulfate and chloride (USEPA 300.1), total nitrate + nitrite (USEPA 353.2), total phosphorus (SM4500‐PI[F]), total Kjeldahl nitrogen (USEPA 351.2), and total organic carbon (SM5310, C‐2000). Third, stormwater samples were analyzed for nutrients (total nitrogen, nitrate/nitrite‐N [NO_*x*_], ammonium‐N [NH_4_^+^], and total phosphorus) by the USEPA Mid‐Continent Ecology Division by standard methods (American Public Health Association & Water Environment Federation, 2005) and for anions (Br^−^ and Cl^−^ [USEPA, 2007] by Legend Technical Services).

#### Chemical contaminants: Organic chemicals

The concentrations of pyriproxyfen spiked into wetland waters used for laboratory exposures were confirmed by gas chromatography–tandem mass spectrometry (method WAU‐100) at the Minnesota Department of Agriculture Laboratory. Stormwater samples were analyzed for pyriproxyfen and a large suite of organic contaminants by the US Geological Survey's (USGS's) National Water Quality Laboratory; see Supporting Information, Table S3, and Fairbairn et al. [2018] for details). Briefly, analytical chemistry methods included USGS methods for approximately 400 CECs and other organics including pesticides, pharmaceuticals, and commercial‐residential products (Furlong et al., 2014; Sandstrom et al., 2016; Zaugg et al., 2006).

#### Chemical contaminants: Metals

Stormwater samples were analyzed for trace metals (Ag, As, Ba, Cd, Cr, Cu, Fe, Hg, Ni, Pb, Se, Zn) with USEPA Method 200.8 using inductively coupled plasma‐mass spectrometry by Legend Technical Services (USEPA, 1994).

#### Phenotype bioassays of stormwater samples

Bioassays included phenotyping for reproduction and survival, as described (Westerhoff et al., 2018). Throughout the experiment, all beakers were inspected daily for mortality, with dead animals removed immediately. With the onset of reproduction, the total number of neonates was counted daily. Neonates were removed from beakers and sexed under 10× magnification as males spotted enlarged antennule (Ebert, 2005).

### Analysis of gene expression

#### RNAseq procedures

Strand‐specific messenger RNA libraries were created for each total RNA sample of pooled daphnia (*n* = 5 individual neonates per replicate sample; five groups per treatment). Illumina HiSeq 2500, Ver 4, chemistry in high‐output mode to obtain 10 million reads per library, 50‐bp paired end (2 × 50), was employed.

#### RNA data processing

Raw sequences were evaluated via FastQC (Andrews, 2010) to determine the baseline quality of the sequencing results. To improve these overall statistics, reads were trimmed to remove Illumina‐specific adapter sequences and low‐quality sequences or sequence tails using the Trimmomatic software (Bolger et al., 2014). FastQC was run again to verify that only high‐quality sequences were retained. High‐quality sequences were then mapped to *D. magna* reference transcripts using Kallisto (Bray et al., 2016), a kmer‐based mapper. Kallisto produces a matrix of read counts with transcripts along one axis and samples along the other. To produce a data set for direct differential expression analysis, each cell of this matrix was rounded to the nearest integer value to be compatible with the DESeq 2 algorithm (Love et al., 2014; an R Package available through Bioconductor). To produce a data set to identify minimal gene signatures separating conditions, each read count was divided by the total reads counted for that sample, producing a fractional matrix. All genes that showed no variance in any one data set were removed from analysis for all data sets to ensure cross‐data set comparability. Initial comparisons indicated a modest batch effect between the wetland and stormwater samples. A linear model was therefore used to identify and remove batch variance that may have been a result of employing two different genetic backgrounds for each study.

### Statistical analyses

#### Pyriproxyfen concentration–response analysis

The LOEC was determined by comparing the percentage of male neonates produced in each treatment level to controls using Wilcoxon's rank‐sum pairwise testing. Concentration–response modeling was performed in R (R Foundation for Statistical Computing, 2020) with the drc package v3.0‐1 (Ritz et al., 2015) to determine effective concentrations at *x*% (EC*x*) for the increased number of male neonates with pyriproxyfen concentration. A binomial two‐parameter log‐logistic model was used to estimate slope and intercept; the upper and lower limits of the model were fixed to 0 and 1, respectively (Ritz et al., 2015).

#### Analysis of the DEG and gene ontology enrichment analysis

To identify DEGs between distinct conditions based on a reference set of 36,992 transcripts DeSeq 2 was used. A DEG was a gene whose expression was >log 2‐fold above or below its respective control at a false discovery rate–adjusted *p* value of 0.05 (*p*_adj_ < 0.05). Several sets of DEGs were produced for subsequent analysis. We employed three methods to analyze global gene expression by treatment group. Among DEGs, enrichment of gene ontology (GO) terms was calculated via a standard hypergeometric test with Bonferroni multiple‐hypothesis correction.

#### Heat mapping

Heat maps were created to highlight pairwise correlation between samples and to show the clustering of selected DEGs via logistic regression and least absolute shrinkage and selection operator (LASSO). Pairwise correlations were calculated using the base R “cor” function and plotted using the base R “heatmap” function. The logistic regression selected genes were plotted using the “ComplexHeatmap” package available through Bioconductor.

#### Principal components analysis

Principal components were calculated using the “prcomp” function in base R with default parameters.

#### Machine learning/logistic regression

The “cv.glmnet” (Friedman et al., 2010) function of the “glmnet” R package was used to perform logistic regression with LASSO regularization and 10‐fold cross‐validation on each of the sample sets. The sample versus gene matrix was centered and scaled on a per sample basis. The response vectors varied to select genes associated with different conditions. These included “pyriproxyfen,” “Location,” and “Category” information from the wetland samples and “Season” and “Site Type” for the stormwater samples. Repeated label randomization was used as a secondary check of the predictive power of the models.

#### Canonical redundancy analysis

Canonical redundancy analysis (RDA) is useful to identify relationships between multiple predictor and multiple response variables while removing redundant effects of covariates (Oksanen, 2015). The RDA and associated plotting of stormwater sample data was performed in R (R Foundation for Statistical Computing, 2020) with the vegan (Oksanen et al., 2019) and adespatial (Dray et al., 2020) packages. Predictor variables comprised individual in situ parameters (e.g., IESF treatment status, developed land‐use percentages), chemicals (e.g., Ba, Cl), and chemical classes (e.g., agricultural herbicides, polycyclic aromatic hydrocarbons [PAHs]) as ranked concentrations, with each chemical or chemical class as a separate predictor. Predictors were structured as ranked concentrations (for individual chemicals) or as the sum of ranked concentrations (for chemical classes). Response variables included *D. magna* phenotype parameters (both RDA models; i.e., survival, reproduction, male offspring) and gene transcripts that were retained as significant in the various logistic regression models of stormwater, wetland, and pyriproxyfen exposure groups (first RDA model) and stormwater exposure groups (second RDA model). All variables were Hellinger‐transformed for RDA (Oksanen, 2015). Model selection was based on the stepwise forward addition procedure and the optimized Akaike's information criterion.

#### Analysis of pyriproxyfen as an internal standard for gene expression in complex matrices

Four DEG sets were produced to evaluate pyriproxyfen as an internal standard for gene expression in the wetland studies: DEG1, wetland water versus laboratory water without pyriproxyfen addition; DEG2, pyriproxyfen‐treated versus control laboratory water; DEG3, pyriproxyfen‐treated versus control wetland waters; and DEG4, wetland water versus laboratory water with pyriproxyfen addition. We then employed both unsupervised and supervised processes to evaluate gene expression. For the unsupervised process, DEG2 and DEG3 sets were intersected to identify DEGs common to both conditions and thus reflective of pyriproxyfen‐specific effects. We then assessed whether these genes may be functionally involved with a mode of action relevant to pyriproxyfen, MfR agonist with subsequent hemoglobin production (Rider et al., 2005) and sex determination (Olmstead & LeBlanc, 2003). For the supervised process, all DEG sets were probed for the presence of DEGs associated with pyriproxyfen's mode of action under the hypothesis that only DEG Sets 2–4 would contain such DEGs. If DEGs associated with pyriproxyfen's mode of action were present in all treatment groups in which pyriproxyfen addition was included, it was considered evidence for a working internal standard in laboratory water and complex matrices like wetland waters.

## RESULTS AND DISCUSSION

### Experiment 1, laboratory validation: Determination of an effect concentration for male neonate production in laboratory‐reared *D. magna* by pyriproxyfen exposure

**Figure 2:**
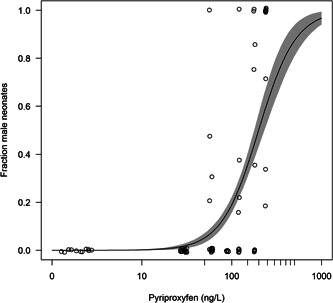
Increased proportion of male *Daphnia magna* neonates with nominal pyriproxyfen exposure concentration. Data points (*n* = 40 per exposure concentration) have been jittered in the *x* and *y* directions. Values <10 ng/L pyriproxyfen are 0 ng/L (nominal). Gray shading indicates 95% confidence intervals about the mean.

The proportion of males increased with pyriproxyfen concentration in laboratory waters (Figure 2) and resulted in a LOEC of 119 ng/L and EC10, EC25, and EC50 values of 70.1 (standard error of the mean [SEM] = 5.0), 118.3 (SEM 5.7), and 199.5 (SEM 13.3) ng/L, respectively. Although more male neonates were produced at a treatment level of 59 ng/L compared to controls (*p* = 0.008), male neonates were not produced at every treatment level until the concentration reached 119 ng/L. Nominal EC*x* values appear to have been higher than measured EC*x* values (Supporting Information, Figure S1, adjusted *R* ^2^ = 0.78, *β*_1_ = 0.47, *p* < 0.0001), but because we only measured pyriproxyfen concentrations in stock solutions, we report nominal EC50 values. The nominal EC50 of 199.5 ng/L is higher than previously reported values. However, using the regression equation shown in Supporting Information (Figure S1) to adjust nominal concentrations to estimated concentrations, we obtained EC10, EC25, and EC50 values of 51.1 (SEM 2.9), 75.7 (SEM 2.7), and 112.1 (SEM 5.6) ng/L, respectively; these values concur with previously reported values. An EC50 of approximately 55 ng/L was reported by Matsumoto et al. (2008) using 13‐day‐old daphnids exposed for 24 h, while an EC50 of approximately 100 ng/L was found after 21 days of exposure by Tatarazako et al. (2003). Our data are consistent with what is known about the biology of sex determination in daphnids. For example, the timing of juvenile hormone or juvenile hormone analog exposure is critical (Abe et al., 2015; Kato et al., 2010). Although Tatarazako et al. (2003) exposed <24‐h‐old daphnids for 21 days and we exposed the same age class for 16 days, each study likely covered the critical sex determination exposure period (7–8 h prior to ovulation) given the similar EC50s between their study (100 ng/L) and ours (112.1 ng/L, adjusted).

### Experiment 2, field applications, wetlands: Evaluation of gene expression in laboratory‐reared *D. magna* exposed to water collected from wetlands of varying quality, with and without pyriproxyfen addition

#### Detection of a pyriproxyfen gene expression signal in complex mixtures

The detection of stressor‐responsive genes that are informative of specific downstream physiological and organismal effects in *D. magna* exposed to complex aqueous matrices can be challenging if the stressors interact at the level of gene expression. To address this concern, we incorporated the well‐studied juvenile hormone analog pyriproxyfen into our studies (Matsumoto et al., 2008; Olmstead & LeBlanc, 2003). The effect of wetland waters with and without pyriproxyfen addition was examined to determine if gene expression due to pyriproxyfen exposure in laboratory‐reared *D. magna* was detectable in environmental waters that potentially contain numerous bioactive chemicals. There were 97 DEGs (*p*_adj_ < 0.05, log fold change > |2.0|; 81 up, 16 down) from exposure to wetland waters compared to laboratory waters (DEG Set 1, reflecting no pyriproxyfen additions). Addition of pyriproxyfen at EC25 (119 ng/L) to laboratory waters resulted in 1487 (514 up, 973 down) DEGs compared to controls (DEG Set 2), while the addition of pyriproxyfen to wetland waters resulted in 230 DEGs (103 up, 127 down) compared to unspiked wetland waters (DEG Set 3). A comparison of gene expression from exposure to pyriproxyfen in wetland waters compared to exposure to pyriproxyfen in laboratory waters resulted in a finding of 1180 DEGs (111 up, 1069 down; DEG Set 4; Supporting Information, Table S4). There were 43 DEGs in common between DEG Sets 2 and 3 (“intersection gene set”; Supporting Information, Table S5). Of the 43 genes (15 had annotations), none appeared to be functionally associated with pyriproxyfen's mode of action. The full count of overlapping DEGs can be found in Supporting Information, Figure S2.

Although the intersection gene set did not indicate the occurrence of identifiable pyriproxyfen responsive genes, we did detect a gene in DEG Set 2 that may play a role in an adverse outcome pathway (AOP) currently being developed (Song & Tollefsen, 2021). This AOP, *Juvenile hormone receptor agonism leading to male offspring induction associated population decline*, describes a pathway that is initiated by the interaction of juvenile hormone (methyl farnesoate in crustaceans) or a juvenile hormone analog such as pyriproxyfen with methoprene‐tolerant (Met) receptor (which we refer to as *MfR* per LeBlanc et al. [2013]), followed by up‐regulation of doublesex1 (*DapmaDsx1*), induction of the male reproductive tract, and subsequent male‐dominated offspring (presumably leading to population decline). In DEG Set 2, the gene set arising from daphnids exposed to pyriproxyfen in laboratory water, doublesex‐ and mab‐3‐related transcription factor 3 (gene Dapma7bEVm005257, which maps to daphplx:hxJGI_V11_22526 and is a close match to Doublsex1 in the *Daphnia pulex* genome) was log fold change 2.55 higher in pyriproxyfen‐exposed compared to unexposed daphnids (*p*_adj_ < 0.0001). Somewhat surprisingly, this gene was also a DEG in DEG Set 1 in which pyriproxyfen exposure was not used. Given that pyriproxyfen is registered (USEPA, 2007, 2018) and used (CDC, 2020) for larval mosquito control and has been successful in controlling mosquitos (Hustedt et al., 2020), it is possible it was used in the study locations and thus present in sampled waters. However, it is more likely that another juvenile hormone analog like methoprene was in wetland waters in Minnesota because it is used in that location (Metropolitan Mosquito Control Center, 2022). The presence of either chemical could have stimulated the up‐regulation of Doublesex1, but we did not analyze for pyriproxyfen or other juvenile hormone analogs in wetland waters. This gene is a member of the doublesex/male‐abnormal‐3 related transcription factor gene family (Picard et al., 2015), which is involved with sex determination and sex differentiation. Of the five known genes in this family, only Doublesex1 is known to play a role in sex determination (Kato et al., 2011). Conflicting with this conclusion is the lack of detection in DEG Sets 3 and 4 (pyriproxyfen added) but detection in DEG Set 1 (no pyriproxyfen). However, the clearest signal was found in DEG Set 2 in which pyriproxyfen was added to laboratory control waters. Additional DEGs of interest were associated with hemoglobin (e.g., di‐domain hemoglobin precursor, Dapma7bEVm001221). Terpenoid hormones and juvenile hormone analogs such as pyriproxyfen have been shown to increase hemoglobin and hemoglobin genes in exposed daphnids (Eytcheson & LeBlanc, 2018; Rider et al., 2005). It has been hypothesized (Rider et al., 2005) that activation of a methyl farnesoate pathway can stimulate up‐regulation of genes responsible for sex determination (Doublesex1; Kato et al., 2011) as well as hemoglobin production (*Dhb2*; Gorr et al., 2004). We detected genes associated with both pathways (hemoglobin and sex determination) in DEG Sets 1 (wetland vs. laboratory water without pyriproxyfen) and 2 (pyriproxyfen vs. control in laboratory water), whereas only genes associated with hemoglobin production were found in DEG Sets 3 (pyriproxyfen vs. control in wetland waters) and 4 (wetland vs. laboratory water with pyriproxyfen). Therefore, it is difficult to conclude the presence of a consistent pyriproxyfen‐stimulated gene signal in our data. The wetland waters may have contained MfR agonists and/or antagonists, which can presumably interact with pyriproxyfen, highlighting the complex nature of gene expression analysis of environmental waters. The lack of a fully annotated *D. magna* genome also complicates the interpretation of our data given that many uncharacterized proteins were DEGs that may or may not clarify which pathways were affected by pyriproxyfen exposure in laboratory and wetland waters. Other complicating factors include the use of a different life stage for gene expression studies (0–4 days) compared to the pyriproxyfen dose–response studies (0–16 days) and a reduced sensitivity to pyriproxyfen treatment in the younger daphnids; the younger life stage is likely less sensitive than adults to pyriproxyfen treatments (Abe et al., 2015; Kato et al., 2010). That is, it is possible that our EC50 of 112.1 ng/L for male neonate production (i.e., effective at influencing expression of genes involved with sex determination) determined with 0‐ to 16‐day‐old daphnids may be lower than what is required to stimulate a similar gene expression response in 0‐ to 4‐day‐olds. Nevertheless, our findings do suggest the presence of pathways activated by Met receptor stimulation via pyriproxyfen or other juvenile hormone analogs. Furthermore, global gene expression analysis indicated a unique response to each treatment.

#### Detection of a water source–specific gene expression signature

**Figure 3:**
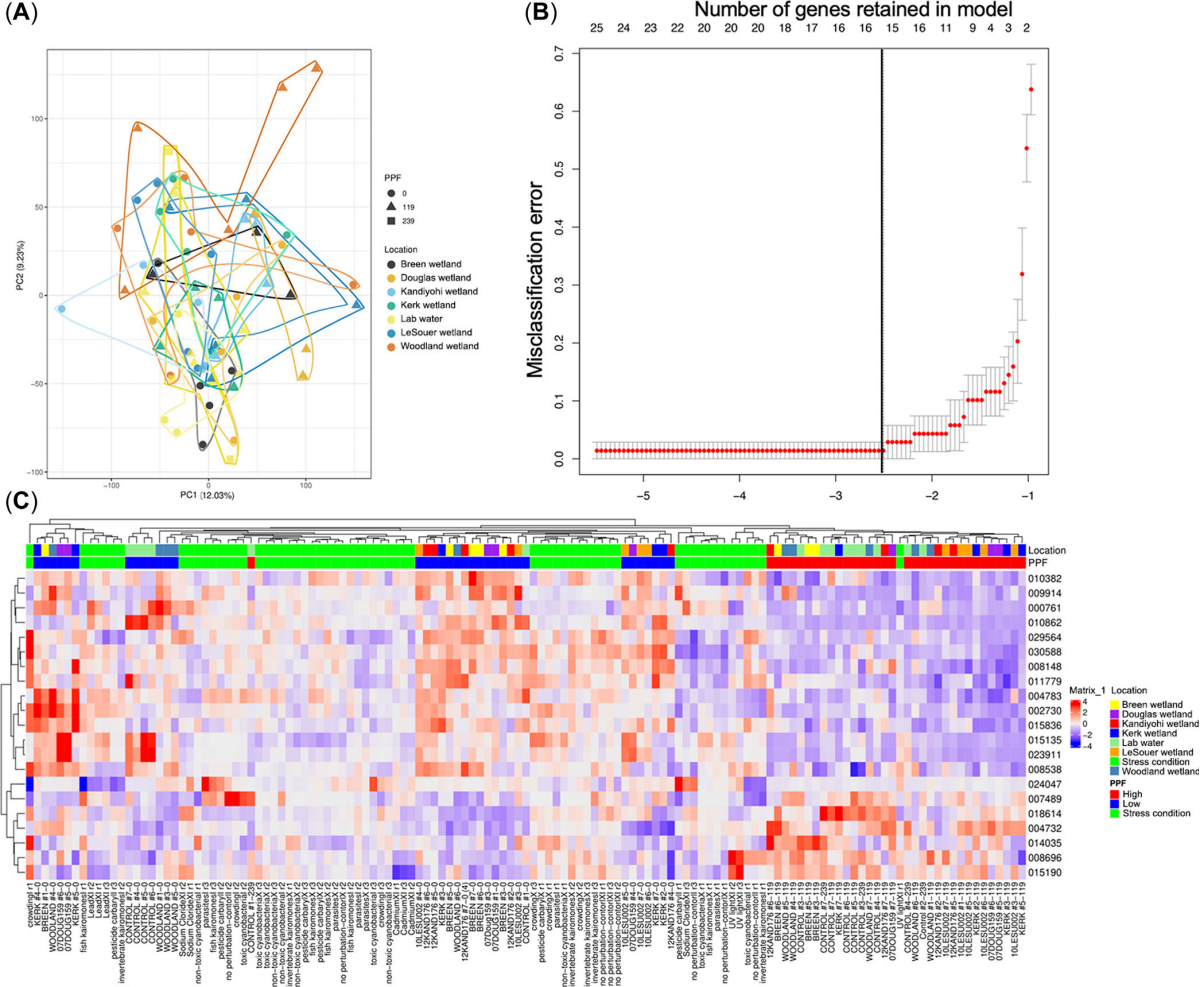
Influence of pyriproxyfen (PPF) and wetland sample type on gene expression in *Daphnia magna*. (**A**) Principal component analysis of gene expression data by wetland location and PPF exposure concentration (nanograms per liter). Outlines are drawn to encompass data that are labeled identically. (**B**) Misclassification error for the wetland PPF spike‐in. The lower *x*‐axis is the lambda parameter setting the complexity of the model, and the upper *x*‐axis is the number of genes remaining in the model at a given complexity. The *y*‐axis is the misclassification error, and the data points (±SEM) describe the error for progressively less complex models. The vertical line represents both the highest‐quality and the least complex model because these models overlapped. (**C**) Heatmap of PPF model–selected genes. Gene identifiers are truncated to six digits by removing the preceding “Dapma7bEVm.” The top bar provides sample annotations with wetland location above PPF levels. The full names of the samples are listed at the bottom (note, 12KAND176 is Kandiyohi wetland). The values on the heatmap range from red (high expression) to blue (low expression). The data displayed for each gene have been variance‐scaled and mean‐centered across the samples. PC = principal component.

Principal component (PC) analysis (PCA) indicated that gene expression was most strongly related to pyriproxyfen exposure rather than IBI and wetland location. A PCA with pyriproxyfen and location labels indicated that PC1 was responsible for 12.03% and PC2 9.23% of the variation. Samples that received pyriproxyfen additions at 239 ng/L tended toward the right side of the plot (*p* < 0.021, sample reassortment over 10,000 iterations with rank‐sum analysis; Figure 3A). Further, more samples treated with 119 ng/L pyriproxyfen plotted on the right (*n* = 19) compared to the left (*n* = 10) side of Figure 3A. There was no clear separation along either axis by IBI category. Furthermore, machine learning identified a logistic regression model containing 16 genes (“modeled gene set”; Table 1) that had strong predictive power for pyriproxyfen status, with a misclassification error <3% (error expected by random chance of 50% and a cutoff of 30.4%, *p* < 0.05; Figure 3B) and showed strong clustering by pyriproxyfen condition (0 vs. 119 ng/L addition; Figure 3C). Thirteen of the genes were expressed at low levels, while the remaining three were at high levels. Gene ontology enrichment for the gene set identified was largely proteolysis‐based, with serine‐type and cysteine‐type endopeptidases being the principle GO terms (Table 1). Lastly, there were two genes (Dapma7bEVm010862 [bestrophin] and Dapma7bEVm023911 [uncharacterized protein, GO serine‐type endopeptidase]) that were common to both the intersection gene set as well as the modeled gene set. Seven related GO terms were significantly (*p* < 0.05) enriched (Supporting Information, Table S6); of note, heme binding (GO:0020037) was an enriched term, potentially indicative of a response to pyriproxyfen (Rider et al., 2005).

**Table 1:** *Daphnia magna* genes responsive to pyriproxyfen treatment as identified by logistic regression

Transcript/gene ID	Gene name	Gene ontology
Dapma7bEVm015836t1	Uncharacterized protein	NA
Dapma7bEVm010862t1	Bestrophin (80%D)	NA
Dapma7bEVm003467t1	Tudor domain‐containing protein (74%M)	
Dapma7bEVm004783t1	Tripartite motif‐containing protein 32,EC:6.3.2.‐/sw (100%D)	GO:0004252/GO:serine‐type endopeptidase activity GO:0006508/GO:proteolysis
Dapma7bEVm015135t1	Uncharacterized protein (98%C)	
Dapma7bEVm008148t1	Uncharacterized protein (67%A)	
Dapma7bEVm023911t1	Uncharacterized protein (63%D)	GO:0004252/GO:serine‐type endopeptidase activity GO:0006508/GO:proteolysis
Dapma7bEVm024047t1	Conserved protein (97%C)	
Dapma7bEVm011779t1	Uncharacterized protein (86%A)	
Dapma7bEVm007489t1	Complement C1q tumor necrosis factor–related protein 3/sw (57%H)	
Dapma7bEVm029564t1	Uncharacterized protein (100%A)	
Dapma7bEVm009914t1	Deoxyribonuclease i (94%D)	GO:0003676/GO:nucleic acid binding GO:0016787/GO:hydrolase activity GO:0046872/GO:metal ion binding
Dapma7bEVm015190t1	Puff‐specific protein Bx42 (100%P)	GO:0000398/GO:mRNA splicing, via spliceosome GO:0005681/GO:spliceosomal complex
Dapma7bEVm010382t1	Isocitrate dehydrogenase [NADP], mitochondrial (99%D)	GO:0016616/GO:oxidoreductase activity, acting on the CH‐OH group of donors, NAD or NADP as acceptor
		GO:0055114/GO:oxidation‐reduction process
Dapma7bEVm030588t1	Uncharacterized protein	
Dapma7bEVm008538t1	Uncharacterized protein (100%D)	GO:0003922/GO:GMP synthase (glutamine‐hydrolyzing) activity; GO:0005524/GO:ATP binding; GO:0006164/GO:purine nucleotide biosynthetic process; GO:0006177/GO:GMP biosynthetic process

NA = not available; GO = gene ontology; mRNA = messenger RNA; NADP = nicotinamide adenine dinucleotide phosphate; NAD = nicotinamide adenine dinucleotide; GMP = guanosine monophosphate; ATP = adenosine triphosphate.

A wetland quality (IBI category) model trained on wetland data excluding pyriproxyfen‐exposed and laboratory water samples contained 19 genes (Supporting Information, Table S7) and generated a modest predictive power with a misclassification rate of 31%–37% (random error of 50%, cutoff of 44%; *p* < 0.05; Supporting Information, Figure S3). The heat map (Supporting Information, Figure S4) indicated a low but discernable degree of clustering of gene expression by wetland quality (high IBI/quality wetlands included Kerk, Douglas, and Kandiyohi; Supporting Information, Table S1) even with the inclusion of pyriproxyfen‐spiked data that the model had not seen previously. This observation suggests that the observed gene pattern shows considerable independence from the pattern separating the pyriproxyfen and non‐pyriproxyfen samples. The laboratory water controls clustered with both high and low IBI samples. The GO terms for these genes point to regulation of RNA expression as a common function. No genes were identified with a wetland location model trained on wetland data excluding pyriproxyfen‐exposed samples, with misclassification error of 58% (error expected by random chance of 63%). It is not surprising that wetland location alone was not a significant factor in gene expression patterns given that there was no replication and, thus, statistical power was low. It is also possible that chemical differences were less associated with the specific wetland than they were with other factors such as environmental setting (e.g., urban vs. agricultural) or season. However, we designed the wetland sampling to assess the effects of IBI/wetland quality on *D. magna* gene expression rather than environmental setting. It is not possible to link our gene expression results to specific chemicals or physical factors because only herbicides were analyzed, and none were detected. In addition, there were differences in water quality parameters across wetland waters that did not pattern with wetland quality classification and therefore may have confounded any wetland quality classification effect (Supporting Information, Table S1). However, water quality parameters did exhibit patterns by quality classification in that “poor”‐quality wetlands had higher chloride, phosphorus, sulfate, total organic carbon, and Kjeldahl N levels than “good”‐quality wetlands. Temperature, pH, and dissolved oxygen were similar across wetland types.

The IBI is used as a means of assessing water quality by many organizations, but it does not allow for the identification of causative chemical factors responsible for IBI differences across sites because it is measured at the community level (Genet & Bourdaghs, 2006). Although gene expression is not an indicator of reduced biological integrity, it may be used to indicate the potential for differences in community indices while providing specific bioactivity information not otherwise available. With further study, such bioactivity information may be used, perhaps, intermittently in place of the IBI, if IBI and gene expression patterns each indicate the presence of a stressor or stressors. Gene expression results may also be used in concert with IBI measurements to increase the chances for detection of specific bioactivities that may be traceable to specific chemical classes or chemical mixtures while also analyzing the community integrity at a site. The opportunity to detect chemical‐ or mixture‐specific bioactivities is especially useful when the stressor(s) is unidentified, chemical analysis of all responsible or potentially responsible compounds is too expensive, and a more rapid assay response time is desired compared to observing slower changes in IBI over time. Lastly, it is also possible to generate a “transcriptomic point of departure” (tPOD) by serially diluting wetland waters and evaluating gene expression using a short‐term assay (<20 days, as in the present study or as in Abe et al. [2015]), similar to what is currently being done for chemical toxicity (Alcaraz et al., 2021). Such a tPOD can be used to track water quality improvements while providing detailed biological response information potentially traceable to chemicals with known modes of action in a format similar to WET testing. However, a cautionary note should be expressed such that, just as in the present study, the *D. magna* transcriptome is insufficiently annotated, so it is not possible to fully infer gene functions and biological pathways affected by chemical or other factors associated with differences in wetland IBI.

### Experiment 3, field applications, stormwater: Exposure of laboratory‐reared *D. magna* to samples taken across three seasonal events from stormwater management infrastructural locations that differed by catchment position and presence of an IESF SCM

#### *D. magna* gene expression patterns in response to stormwater exposures

The purpose of exposing *D. magna* to a range of stormwater sample types was to determine if gene expression patterns could be used to distinguish sample origin. We specifically investigated if and what parallel patterns emerged among stormwater chemistry, *D. magna* gene expression, and *D. magna* phenotype parameters based on stormwater sample season and/or site type. A comparison of spring versus early summer stormwater samples indicated 436 DEGs (131 up, 305 down, DEG Set 5), while that of spring versus late summer indicated 347 DEGs (80 up, 267 down) and that of early summer versus late summer indicated 213 DEGs (71 up, 142 down). Comparison of IESF‐untreated versus IESF‐treated samples indicated only two DEGs (both up‐regulated, uncharacterized proteins). Among the DEGs, GO term enrichment indicated that adenosine triphosphate binding, protein binding, and zinc ion binding were in response to differences in season of sample collection (Supporting Information, Table S8). Early summer versus late summer indicated the highest number of enriched terms, which corresponds to the largest difference in the types of chemicals detected as well (Westerhoff et al., 2018).

**Figure 4:**
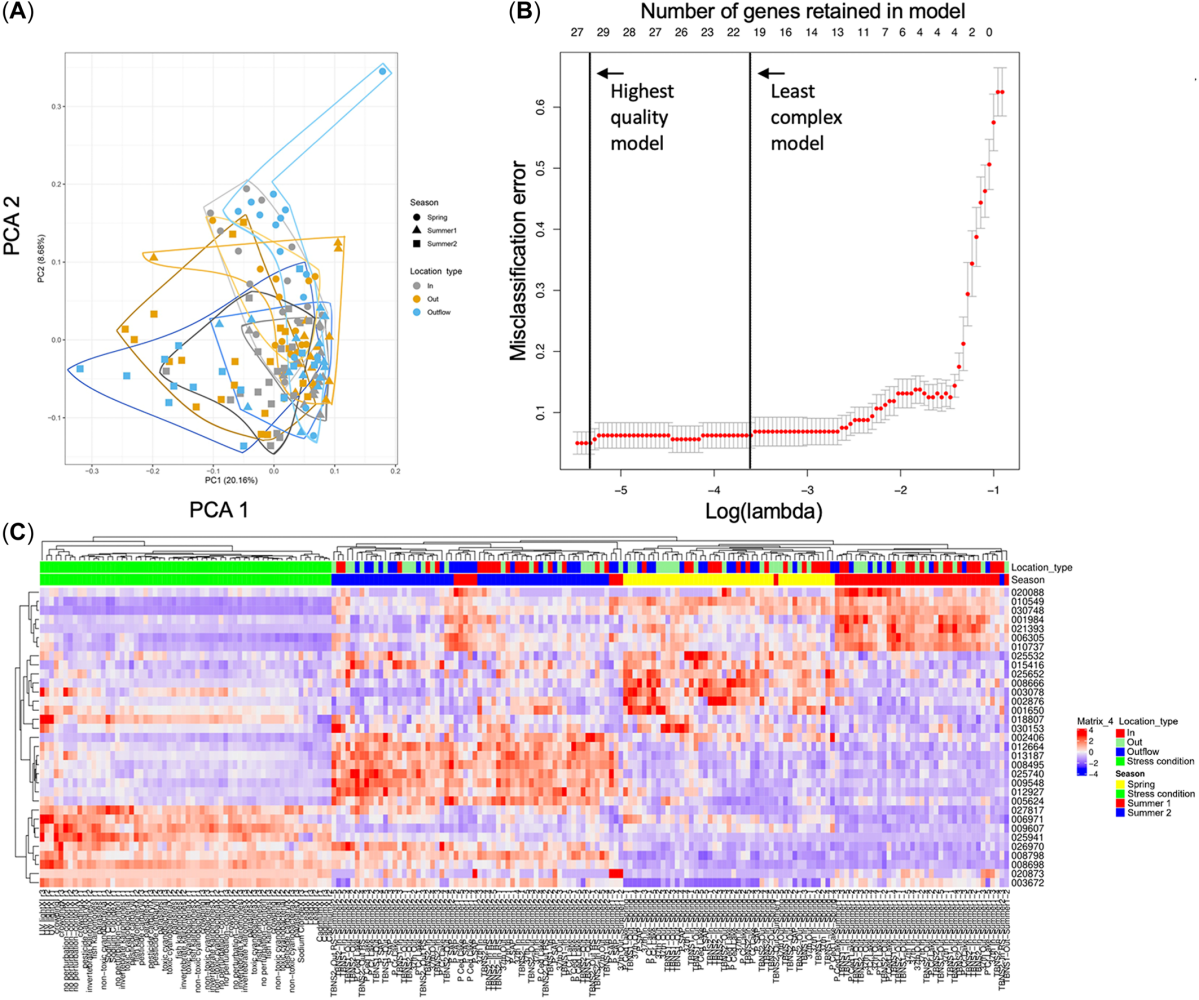
Influence of stormwater sample type and season on gene expression in *Daphnia magna*. (**A**) Principal component analysis of gene expression data by stormwater sampling season (Summer1 = early summer; Summer2 = late summer) and site type (In = pretreatment; Out = posttreatment; Outflow = collection pipe outflow). (**B**) Misclassification error for stormwater seasonal model. The lower *x*‐axis is the lambda parameter setting the complexity of the model, and the upper *x*‐axis is the number of genes remaining in the model at a given complexity. The *y*‐axis is the misclassification error, and the data points (±SEM) describe the error for progressively less complex models as lambda increases. The two vertical lines represent the highest‐quality model and the least complex model within 1 SE of the highest‐quality model. (**C**) Heatmap of stormwater season model–selected genes. Gene identifiers are truncated to six digits by removing the preceding “Dapma7bEVm.” The top bar provides sample annotations with location (site) type above season. The full names of the samples are listed at the bottom. The values on the heatmap range from red (high expression) to blue (low expression). The data displayed for each gene have been variance‐scaled and mean‐centered across the samples. PCA = principal component analysis.

Likewise, a PCA of the stormwater samples (Figure 4A) indicated that gene expression separated more strongly based on season than site type. Principal component 1 accounted for 20.71% of the total variance, with early summer enrichment (“Summer1”) on its higher end and broad separation of late summer (“Summer2”). Principal component 2 accounted for 8.68% of the variance, with all seasons separated, spring enriched at the higher end, and early and late summer enriched at the lower end. Although less prominent, site type separations were evident, for example, IESF‐untreated enrichment on the higher end of PC1.

For stormwater samples, two distinct machine learning logistic regression models were trained, which differentiated based on stormwater site type and season, respectively. The “seasonal” stormwater logistic regression model reflected a misclassification error of 6%–7%, which was excellent overall and significantly better than the expected error (46%) and *p* < 0.05 cutoff (31%; Figure 4B). The model was relatively complex, with 57 genes retained, which were generally related to broad cellular functions (e.g., autophagy, ribosomes, metabolism; Table 2). The corresponding heat map (Figure 4C) clearly separated clusters based on the three seasonal events and indicated groups of genes associated with each. Table 2 lists the genes maintained in the best season model. Transcripts Dapma7bEVm001483t (associated with zwilch, a mitotic checkpoint component) and Dapma7bEVm027817t1 (associated with long chain fatty acid catalysis) were differentially expressed both in the laboratory pyriproxyfen versus control (DEG Set 2) and stormwater season experimental groups; both were significantly down‐regulated in pyriproxyfen‐spiked laboratory waters and in the early summer stormwater samples, which coincided with elevated concentrations of many analyzed pesticides.

**Table 2:** *Daphnia magna* genes responsive to seasonal differences as identified by logistic regression

Transcript/gene ID	Gene name	Gene ontology
Dapma7bEVm018451t1	2‐Hydroxyacylsphingosine 1‐beta‐galactosyltransferase (95%H)	GO:0016758/GO:transferase activity, transferring hexosyl groups; GO:0008152/GO:metabolic process
Dapma7bEVm008077t1	Pancreatic triacylglycerol lipase (90%H)	
Dapma7bEVm001650t1	Uncharacterized protein (58%D)	
Dapma7bEVm006808t1	Lactosylceramide (100%D)	
Dapma7bEVm012269t1	Uncharacterized protein (99%D)	
Dapma7bEVm003078t1	Uncharacterized protein (100%D)	
Dapma7bEVm023563t1	wd‐repeat protein (88%D)	
Dapma7bEVm025532t1	Uncharacterized protein	
Dapma7bEVm005448t1	Cohesin‐subunit (97%D)	
Dapma7bEVm021704t1	Uncharacterized protein (59%D)	
Dapma7bEVm002876t1	CG8483 (100%D)	
Dapma7bEVm025652t1	Uncharacterized protein	
Dapma7bEVm030153t1	Uncharacterized protein (66%R)	GO:0006914/GO:autophagy
Dapma7bEVm001483t1	Zwilch (78%H)	
Dapma7bEVm015465t1	Uncharacterized protein (100%A)	
Dapma7bEVm027904t1	Mitotic checkpoint serine/threonine‐protein kinase BUB1 beta (85%D)	GO:0004672/GO:protein kinase activity; GO:0005524/GO:ATP binding; GO:0006468/GO:protein phosphorylation
Dapma7bEVm018807t1	Uncharacterized protein (75%C)	
Dapma7bEVm013586t1	Uncharacterized protein	
Dapma7bEVm025941t1	Uncharacterized protein	
Dapma7bEVm022658t1	Uncharacterized protein (96%C)	
Dapma7bEVm002406t1	Carbohydrate sulfotransferase (88%M)	
Dapma7bEVm015416t1	Uncharacterized protein (67%A)	
Dapma7bEVm027817t1	Long‐chain‐fatty‐acid–CoA ligase (65%R)	GO:0003824/GO:catalytic activity; GO:0008152/GO:metabolic process
Dapma7bEVm003099t1	Phospholipase DDHD2 (100%D)	GO:0046872/GO:metal ion binding
Dapma7bEVm023721t1	Uncharacterized protein (100%D)	
Dapma7bEVm014048t1	Uncharacterized protein (57%A)	
Dapma7bEVm008640t1	Peptidyl‐tRNA hydrolase PTRHD1 (83%H)	GO:0004045/GO:aminoacyl‐tRNA hydrolase activity
Dapma7bEVm012927t1	Uncharacterized protein	
Dapma7bEVm012664t1	Uncharacterized protein (84%A)	
Dapma7bEVm010382t1	Isocitrate dehydrogenase [NADP], mitochondrial (99%D)	GO:0016616/GO:oxidoreductase activity, acting on the CH‐OH group of donors, NAD or NADP as acceptor; GO:0055114/GO:oxidation‐reduction process
Dapma7bEVm010549t1	NADH dehydrogenase [ubiquinone] 1 beta subcomplex subunit 11, mitochondrial (100%D)	
Dapma7bEVm020260t1	mitochondrial phosphate carrier protein (52%D)	
Dapma7bEVm026970t1	Uncharacterized protein	
Dapma7bEVm014131t1	Uncharacterized protein (71%A)	
Dapma7bEVm025740t1	Uncharacterized protein	
Dapma7bEVm014871t1	Uncharacterized protein	
Dapma7bEVm008495t1	Uncharacterized protein	
Dapma7bEVm005624t1	Pantothenate kinase 2, mitochondrial (76%H)	GO:0004594/GO:pantothenate kinase activity; GO:0005524/GO:ATP binding; GO:0015937/GO:coenzyme A biosynthetic process
Dapma7bEVm030748t1	Uncharacterized protein (100%D)	
Dapma7bEVm018251t1	Uncharacterized protein (71%L)	GO:0004672/GO:protein kinase activity; GO:0005524/GO:ATP binding; GO:0006468/GO:protein phosphorylation
Dapma7bEVm028792t1	Spliceosome‐associated protein CWC15 (80%D)	GO:0000398/GO:mRNA splicing, via spliceosome; GO:0005681/GO:spliceosomal complex
Dapma7bEVm000189t1	Embryonic polyadenylate‐binding protein (100%T)	GO:0003723/GO:RNA binding
Dapma7bEVm008798t1	Tumor necrosis factor alpha–induced protein 8 protein 3 (100%D)	
Dapma7bEVm008931t1	Conserved protein (100%C)	
Dapma7bEVm006305t1	39S ribosomal protein L44, mitochondrial (100%D)	GO:0003723/GO:RNA binding; GO:0004525/GO:ribonuclease III activity; GO:0006396/GO:RNA processing
Dapma7bEVm011371t1	DNA repair protein XRCC2 (100%D)	
Dapma7bEVm010737t1	Peroxisomal biogenesis factor (91%P)	GO:0007031/GO:peroxisome organization; GO:0005779/GO:integral component of peroxisomal membrane
Dapma7bEVm006628t1	Conserved protein (100%P)	
Dapma7bEVm001975t1	Glucose‐6‐phosphatase (83%H)	GO:0003824/GO:catalytic activity; GO:0016020/GO:membrane
Dapma7bEVm030729t1	Cytochrome c oxidase assembly factor (92%D)	GO:0004129/GO:cytochrome‐c oxidase activity; GO:0005739/GO:mitochondrion
Dapma7bEVm021605t1	Uncharacterized protein	
Dapma7bEVm020088t1	rve, Integrase core domain (92%C)	GO:0015074/GO:DNA integration
Dapma7bEVm003672t1	Sodium‐coupled neutral amino acid transporter (100%D)	
Dapma7bEVm014202t1	60S ribosomal protein L23a (27%D)	GO:0003735/GO:structural constituent of ribosome; GO:0006412/GO:translation; GO:0005840/GO:ribosome
Dapma7bEVm021300t1	Uncharacterized protein (66%R)	
Dapma7bEVm014567t1	Uncharacterized protein	
Dapma7bEVm020873t1	60s ribosomal protein l23 (89%D)	GO:0003735/GO:structural constituent of ribosome; GO:0006412/GO:translation; GO:0005840/GO:ribosome

GO = gene ontology; ATP = adenosine triphosphate; CoA = coenzyme A; tRNA = transfer RNA; NADP = nicotinamide adenine dinucleotide phosphate; NAD = nicotinamide adenine dinucleotide; NADH = reduced form of NAD.

As with DEG counts and PCA, season impacted gene expression more consistently than site type. The observed misclassification error of the “site type” model was 55%–57%, which was poor but still significantly better than the expected error (71%) and *p* < 0.05 cutoff (65%). This model retained 18 genes, which are annotated to a number of transcriptional and signaling regulation terms (Supporting Information, Table S9). A heat map (Supporting Information, Figure S5) of these genes’ expression levels by site type primarily distinguished the PIPE‐untreated samples from the IESF‐treated and IESF‐untreated samples. The IESF‐treated and IESF‐untreated samples from the same sampling events often coclustered, which is sensible given their similar source waters and their primary difference being treatment status, which would imply co‐occurrence of some identical chemicals (and thus bioactivities), albeit perhaps at different concentrations.

#### Relationships between gene expression and chemical detection patterns

**Figure 5:**
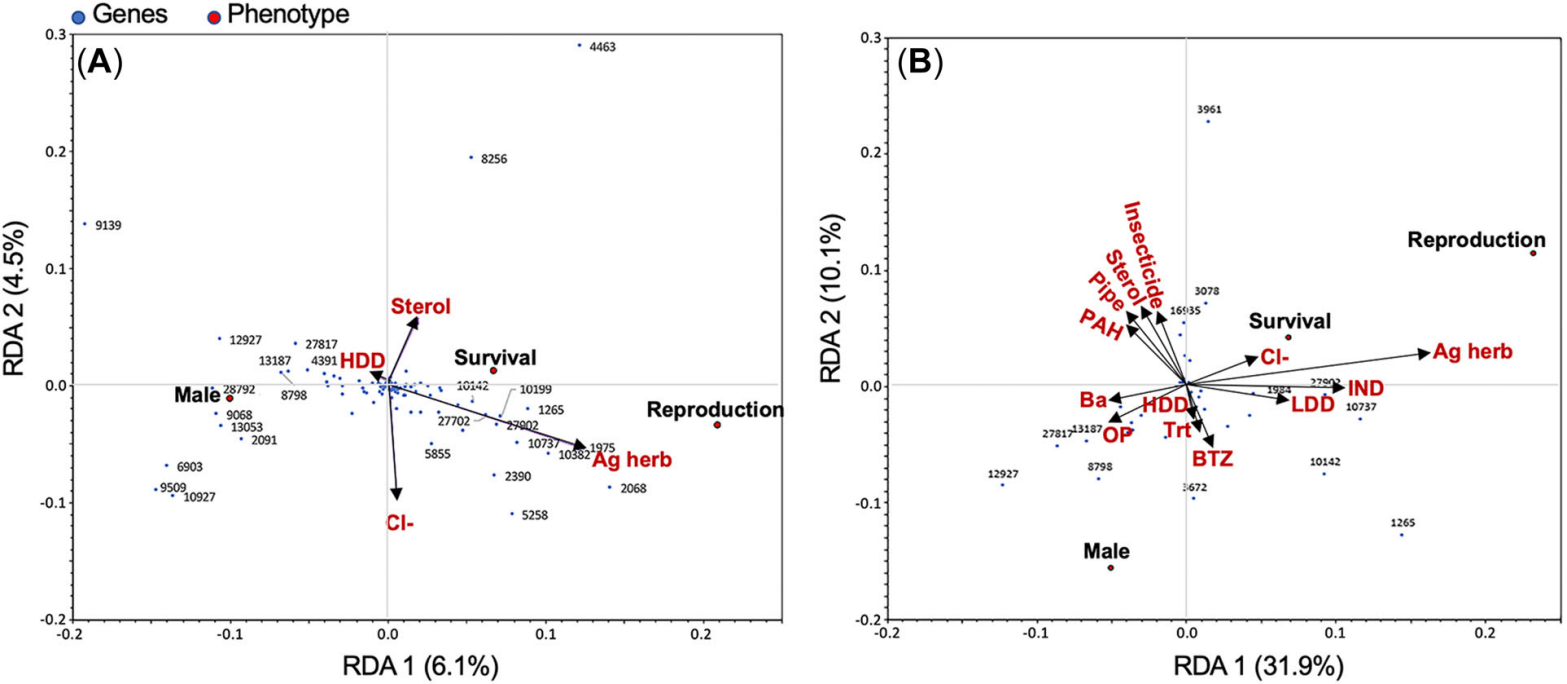
Canonical redundancy analysis (RDA) of differentially expressed *Daphnia magna* genes and phenotypes (response variables, represented as dots, reported in Westerhoff et al. [2018]) following daphnid exposure to stormwater samples based on chemical class and in situ conditions (predictor variables, represented as arrows), with response variables including all differentially expressed genes (DEGs) identified in (**A**) Experiments 1‐3 (variable wetland, pyriproxyfen, and stormwater exposures) and (**B**) Experiment 3 only (variable stormwater exposures). Daphnids (<24 h) were treated with a sample for 96 h, and RNA was extracted for gene expression analysis. Each blue dot represents a DEG, a subset of which are labeled with the corresponding gene identifier truncated to five or four digits (when the five‐digit number begins with a 0). For example, “Dapma7bEVm010549t1” would be labeled as *10549*. In a subsequent experiment, daphnids (<24 h) were treated for 16 days and examined for phenotypic changes. Each red dot indicates a realized phenotypic response. Arrows (red text) indicate chemical classes and in situ conditions previously quantified (Fairbairn et al., 2018) for the stormwater samples of the present study that were identified as significant predictors by RDA. HDD = high‐density development; Ag herb = agricultural herbicides; Urb insect = urban‐use insecticides; Pipe = pipe‐untreated sites; PAH = polycyclic aromatic hydrocarbon; Ba = barium; OP = orthophosphate; BTZ = benzotriazoles; LDD = low‐density development; IND = industrial‐use compounds; Trt = treatment of stormwater [by IESF SCM].

As with its relative influence on gene expression, season exhibited a larger effect than site type on chemical detections. Briefly, in stormwater, 58 pesticides and their degradates were detected at least once; detection frequencies were >25% for 20 and ≥50% for 11 of these. However, pyriproxyfen was not detected (reporting limit = 3 ng/L). An additional 65 organic compounds were detected at least once, along with metals, nutrients, and chloride. Supporting Information, Table S3, describes some general chemical differences among stormwater sample types and seasons, with complete details in Fairbairn et al. (2018). Because linkages of chemical exposures, differential gene expression, and biological outcomes were of interest, we conducted canonical RDA to formally examine the relationships among chemical and in situ predictors and phenotypic and transcriptomic responses in stormwater samples. The RDA biplots (Figure 5) illustrate correlations among various parameters. In Figure 5, the arrows represent predictors retained as significant by RDA, and the points represent responses. The relative direction among parameters indicates the direction of association, while the distance from origin indicates the magnitude. Also see Supporting Information, Table S10 for RDA scores.

First, an RDA that included all DEGs identified among all sample set comparisons (including comparisons with laboratory and wetland water) was completed (Figure 5A). This model had relatively low explanatory power (adjusted *r* ^2^ = 0.207). The first two RDA axes explained 6.1% and 4.5% of the total model inertia, with constrained inertia accounting for only 13.5% of total inertia (meaning that 86.5% of total inertia was explained by unconstrained inertia not associated with any RDA axis). A second RDA (Figure 5B) with DEGs limited to those identified by comparisons among different groups of stormwater samples (i.e., by season and site type) showed improved predictive power (adjusted *r* ^2^ = 0.502), with the first two RDA axes explaining 31.9% and 10.1% of the total model inertia, respectively, and constrained inertia accounting for 53.7% of total inertia. These results are sensible considering that the first RDA model likely had greater “noise” than the second because of the first's inclusion of response DEGs that did not in fact vary significantly among stormwater sample groups, although they had varied significantly among other sample matrices (i.e., laboratory and wetland water).

Certain benzotriazoles, including those detected in stormwater samples in the present study, have been identified as *D. magna* endocrine disruptors based on up‐regulation of transcripts related to molting and vitellogenin, including perturbation of ecdysteroid‐mediated pathways (Giraudo et al., 2017); indeed, Figure 5B indicates a positive association of benzotriazoles with male neonate production. Other chemical classes in Figure 5B did not obviously reflect anticipated patterns. For example, agricultural herbicides may be expected to correlate with reduced *D. magna* survival and reproduction, but Figure 5B indicates the opposite. Likewise, several predictors indicated orthogonal (e.g., urban insecticides, PAHs) or counterintuitive (e.g., chloride) associations with phenotypic outcomes. Finally, although the typically hydrophobic sterol and PAH groups aligned with the PIPE‐untreated site type, in agreement with generalized linear mixed modeling results on a similar sample set (Fairbairn et al., 2018), and corresponded with up‐regulation of several transcripts, they did not evidently track with phenotypic outcomes that would indicate toxicologic responses. One explanation for some observed counterintuitive patterns may be that certain represented predictors were associated with other nonassessed and potentially confounding factors that offset the influence of the assessed predictors. For example, agricultural herbicide profiles were strongest in spring and early summer. Indeed, detected metolachlor, acetochlor, and/or oxyfluofen concentrations in spring samples were ≥10% of their reported chronic invertebrate toxicity benchmarks, with one metolachlor detection (490 ng/L) at half of its benchmark (1 µg/L [USEPA, 2022]). Considering potential toxicological additivity, chronic, sublethal effects may be expected (Nowell et al., 2018). However, it is also plausible that herbicide concentrations were low enough to exert minimal toxicity. Yet, both periods showed increased nutrient concentrations and weaker profiles of other contaminants (e.g., PAHs and flame retardants) that typically occur in greater concentrations during low‐flow (e.g., late summer) conditions. It is possible that any (negative) effects of agricultural herbicides (and other contaminants) on *D. magna* phenotypes were overshadowed by positive effects of an enriched growth medium (Westerhoff et al., 2018).

The RDA results indicated that a number of transcripts corresponded with offspring number and/or male neonate production. For example, in Figure 5B, up‐regulation of transcripts Dapma7bEVm13187t, Dapma7bEVm27817t, Dapma7bEVm08798t, and Dapma7bEVm12927t appear to be negatively correlated with number of offspring and positively correlated with male neonate induction. Other transcripts unlabeled in Figure 5B show similar correlation directions at lesser magnitudes (see Supporting Information, Table S10).

## SYNTHESIS AND CONCLUSIONS

Given the expense of chemical analysis, the problem of missed chemical detections due to inadequate detection limits, lack of specific information that certain chemicals should be measured, and a lack of known toxicity thresholds for most chemicals, our primary goal was to evaluate whether invertebrate transcriptomics may be useful for water quality monitoring. We evaluated the *D. magna* transcriptome in response to three distinct source waters in Minnesota. Laboratory‐reared *D. magna* were exposed to these waters for 96 h because short‐term assays are preferred to more expensive longer‐term assays and because our goal was to ascertain initial and specific gene expression in response to a stressor rather than evaluate the organism's acclimation to that stressor over time. We show that the exposure of laboratory‐reared *D. magna* to a juvenoid hormone mimic, pyriproxyfen, wetland, and stormwaters resulted in unique transcriptomic profiles.

*Daphnia magna* gene expression was different across source waters. Sample misclassification error rates were lowest when assigning a sample to whether it came from a pyriproxyfen‐treated daphnid followed by classification to season, wetland quality, stormwater treatment type, then wetland location. It is not surprising that pyriproxyfen misclassification error rates were lowest because pyriproxyfen is biologically active in invertebrates, stimulating the methyl farnesoate signaling pathway (Olmstead & LeBlanc, 2003). Furthermore, pyriproxyfen treatment occurred in laboratory water, which presumably contains fewer chemicals than environmental waters. The effect of season on gene expression was also consistent, with a misclassification error rate of 6%, a remarkably low error rate given the variety of compounds detected in the urban stormwater samples used in our study (Fairbairn et al., 2018) and the potential for interactive biological effects from the exposure to chemical mixtures (Van Gestel et al., 2016). The effect of sample collection season on gene expression results was most likely influenced by factors associated with the water samples rather than potential circannual rhythms (Pernold et al., 2021) of physiological processes or gene expression profiles because all stormwater exposures occurred in January rather than coinciding with stormwater collection dates. Lastly, daphnid gene expression was also somewhat predictive of the quality of the wetland from which a sample was collected (33% misclassification error). Wetland location was the least likely to be consistently identified by gene expression patterns, with a nonstatistically significant logistic regression and a misclassification error rate of 58%.

Thus, it appears to be possible to use *D. magna* as an indicator of differences in biological activity across source waters. However, although gene expression patterns were unique to each source water and pyriproxyfen exposure up‐regulated Doublesex1 as expected, our understanding of the downstream physiological consequences remains limited because of a poorly annotated *D. magna* genome, a well‐recognized shortcoming relative to vertebrate transcriptomics (Ford & LeBlanc, 2020). Our data therefore demonstrate both the benefits and the challenges of incorporating invertebrate transcriptomics into water quality monitoring efforts.

## Supporting Information

The Supporting Information is available on the Wiley Online Library at https://doi.org/10.1002/etc.5392.

## Disclaimer

The present study is not a product of the US government or the USEPA. The views expressed are the authors' own and do not necessarily represent those of the United States or the USEPA.

## Author Contributions Statement

**Mark D. Jankowski**: Conceptualization; Data curation; Formal analysis; Funding acquisition; Investigation; Methodology; Visualization; Project administration; Resources; Writing—original draft. **David J. Fairbairn**: Conceptualization; Data curation; Formal analysis; Investigation; Methodology; Visualization; Project administration; Writing—original draft. **Joshua A. Baller**: Data curation; Formal analysis; Methodology; Project administration; Resources; Visualization; Writing—review & editing. **Benjamin M. Westerhoff**: Data curation; Methodology; Investigation. **Heiko L. Schoenfuss**: Project administration; Resources; Supervision; Visualization; Writing—review & editing.

## Supporting information

This article includes online‐only Supporting Information.

Supporting information.

Supporting information.

Supporting information.

Supporting information.

Supporting information.

Supporting information.

Supporting information.

## Data Availability

RNA sequencing data are available in the NCBI Gene Expression Omnibus (https://www.ncbi.nlm.nih.gov/geo/query/acc.cgi?acc=GSE208591). Statistical R code are available in GitHub (https://github.com/jankow55/Daphnia-bioassay.git). Other data are available via the Data Repository for University of Minnesota (https://doi.org/10.13020/4hq3-v890).
