# Supplementary material for: Using the Daphnia magna Transcriptome to Distinguish Water Source: Wetland and Stormwater Case Studies
Source: Environ Toxicol Chem. 2022 Aug 9;41(9):2107–23. doi: 10.1002/etc.5392 (PMC9545677; doi:10.1002/etc.5392)
Supplement: Supplementary file 1 — Supporting information. [file ETC-41-2107-s004.docx]

**Supplemental Table S1**. Physical and chemical description of wetlands.

| **Wetland Name** | **Acres** | **Latitude** | **Longitude** | **IBI^1^ Score**  **(category)** | **Date**  **Sampled** | **pH** | **°C** | **Water Chemistry of Wetland Samples (mg/L)** | | | | | | | | |
| --- | --- | --- | --- | --- | --- | --- | --- | --- | --- | --- | --- | --- | --- | --- | --- | --- |
|  |  |  |  |  |  |  |  | **Dissolved**  **Oxygen** | **TSS** | **Alkalinity** | **Chloride** | **NO_3_+NO_2_** | **P** | **SO_4_** | **Kjeldahl N** | **TOC** |
| Woodland | 616.85 | 4989184 | 429218.5 | 35 (poor) | 7/9/2015 | 9.21 | 20.4 | 3.05 | 47 | 180 | 49.2 | <0.05 | 0.21 | 8.97 | 4.74 | 20 |
| Le Souer | 63.46 | 4914248 | 450166.2 | 42 (poor) | 7/9/2015 | 9.02 | 21.1 | 3.61 | 5.2 | 130 | 21.9 | 0.05 | 0.77 | 9.87 | 1.29 | 17 |
| Breen | 29.86 | 4901302 | 435208.5 | 43 (poor) | 7/9/2015 | 7.96 | 20.1 | 4.09 | 5.2 | 170 | 11.0 | <0.05 | 0.20 | 1.43 | 1.11 | 16 |
| Douglas | 4.70 | 5085001 | 317156.6 | 68 (good) | 7/8/2015 | 8.76 | 22.1 | 3.14 | 42 | 100 | 54.6 | 0.05 | 0.10 | <0.50 | 1.73 | 11 |
| Kandiyohi | 3.29 | 5023856 | 326468.6 | 75 (good) | 7/8/2015 | 9.28 | 22.1 | 3.09 | 2.4 | 150 | 0.57 | <0.05 | 0.04 | <0.50 | 0.94 | 10 |
| Kerk | 3.99 | 5025728 | 314126.6 | 81 (good) | 7/8/2015 | 8.27 | 21.4 | 2.97 | 8.0 | 200 | <0.50 | <0.05 | 0.06 | <0.50 | 1.28 | 16 |

Notes,

1, index of biological integrity

**Supplemental Table S2**. Description of stormwater sample locations.

| **Site ID** | **Site type** | **GPS Coordinates** | **Catchment (ha)** | **Catchment Impervious Cover (%)** | **Catchment land uses** |
| --- | --- | --- | --- | --- | --- |
| P1 | PIPE-Untreated | 45.02417437964924,  -93.32392454164072 | 187 | 40% | Residential |
| P2 | PIPE-Untreated | 44.95939451163767,  -93.32726992654355 | 789 | 35% | Residential, industrial, commercial |
| P3 | PIPE-Untreated | 44.95345701908334,  -93.20540793643903 | 1380 | 48% | Industrial, residential, university |
| I1 | IESF-Untreated | 44.97628037026484,  -93.09279298472178 | 14.1 | 46% | Residential, commercial |
| I1T | IESF-Treated | 44.97632970246557,  -93.09271520065793 |  |  |  |
| I2 | IESF-Untreated | 44.97402053045891,  -93.09299951481529 | 17.4 | 24% | Residential, cemetery |
| I2T | IESF-Treated | 44.97399017096408,  -93.09290831970596 |  |  |  |
| I3 | IESF-Untreated | 45.02222972178188,  -93.30457307416711 | 0.486 | 50% | Residential |
| I3T | IESF-Treated | 45.02225436811458,  -93.30462940055816 |  |  |  |

**Table S3**. Summary of stormwater sample chemistry by season and site type (data from Fairbairn et al. 2018).

| **Seasons** | **# CECs Detected** | | **Rank Sum of Chemical Concentrations^a^** | | **Notes: CECs and conventional WQ** |
| --- | --- | --- | --- | --- | --- |
|  | Median | Range | Median | Range |  |
| Spring | 39 | 29-54 | 1087 | 703-1590 | Untreated: agricultural/mixed-use herbicide, fungicide, PPCP, commercial-consumer |
| Early Summer | 32 | 28-49 | 1016 | 817-1550 | Untreated: phosphate (med: 385 µg/L), agricultural/mixed-use/non-ag herbicide & degradate, PPCP |
| Late Summer | 30 | 18-46 | 718 | 423-1315 | Fungicide, mixed-use insecticide, DEET, flame retardant |
| **Site Types** |  |  |  |  |  |
| PIPE-Untreated | 39.5 | 24-54 | 1159 | 790-1296 | PAH/coal tar-related, fungicides |
| IESF-Untreated | 36 | 30-41 | 1052 | 423-1129 | Phosphate (med: 350 µg/L), commercial-consumer, PPCP, sterol |
| IESF-Treated | 28.5 | 18-39 | 711 | 490-1590 | *Vs.* IESF-Untreated samples, IESF-Treated samples generally had lower: phosphate (med: 140 µg/L), PAH, PPCP, flame retardant, commercial-consumer, hydrophobic pesticide |

^a^ This metric was derived by ranking the concentrations of each analyzed CEC over all stormwater samples and then using the sum of ranks of individual CECs in a given sample as the rank-sum score for that sample. Higher concentrations received higher ranks; if non-detect data were present, they received the lowest rank(s) for that CEC.
