# Supplementary figures and images for: Using the Daphnia magna Transcriptome to Distinguish Water Source: Wetland and Stormwater Case Studies
Source: Environ Toxicol Chem. 2022 Aug 9;41(9):2107–23. doi: 10.1002/etc.5392 (PMC9545677; doi:10.1002/etc.5392)

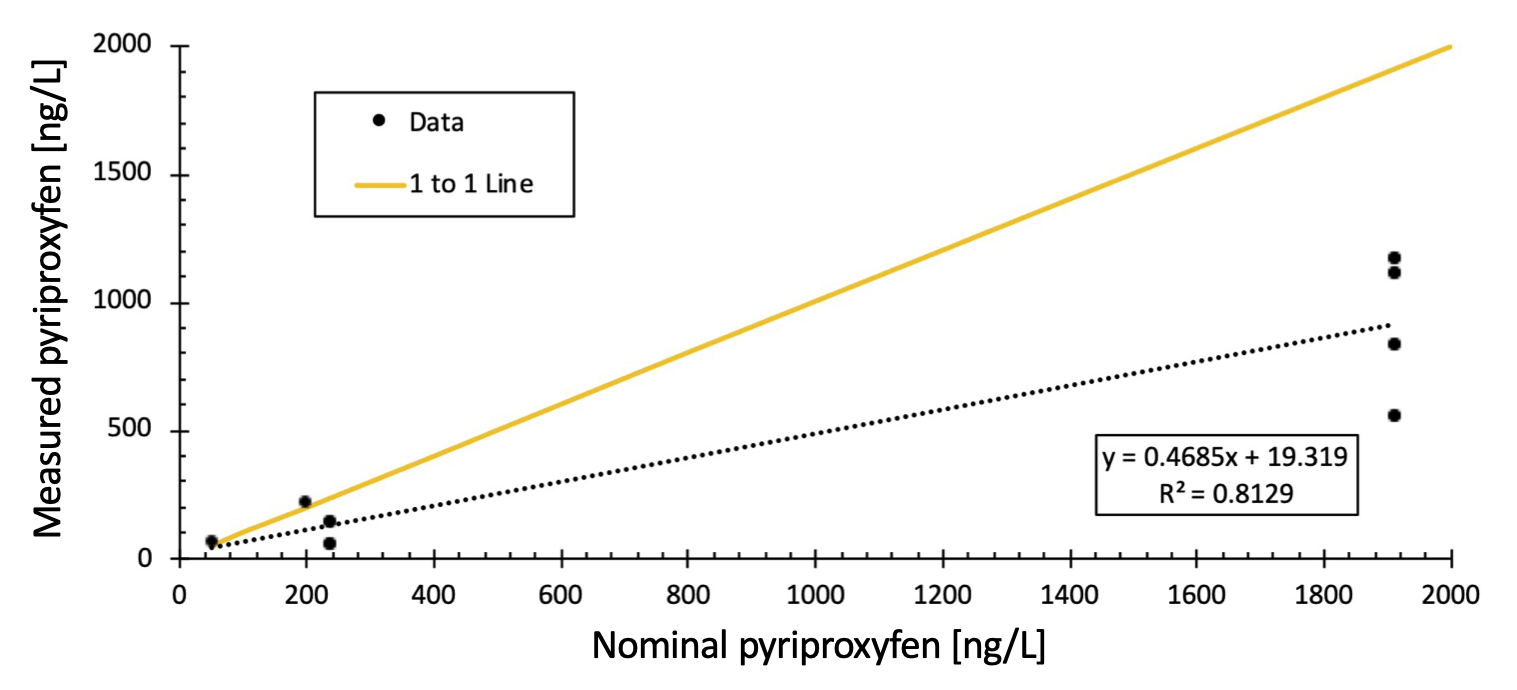

Supplement: Supplementary file 3 — Supporting information. [file ETC-41-2107-s005.tif]

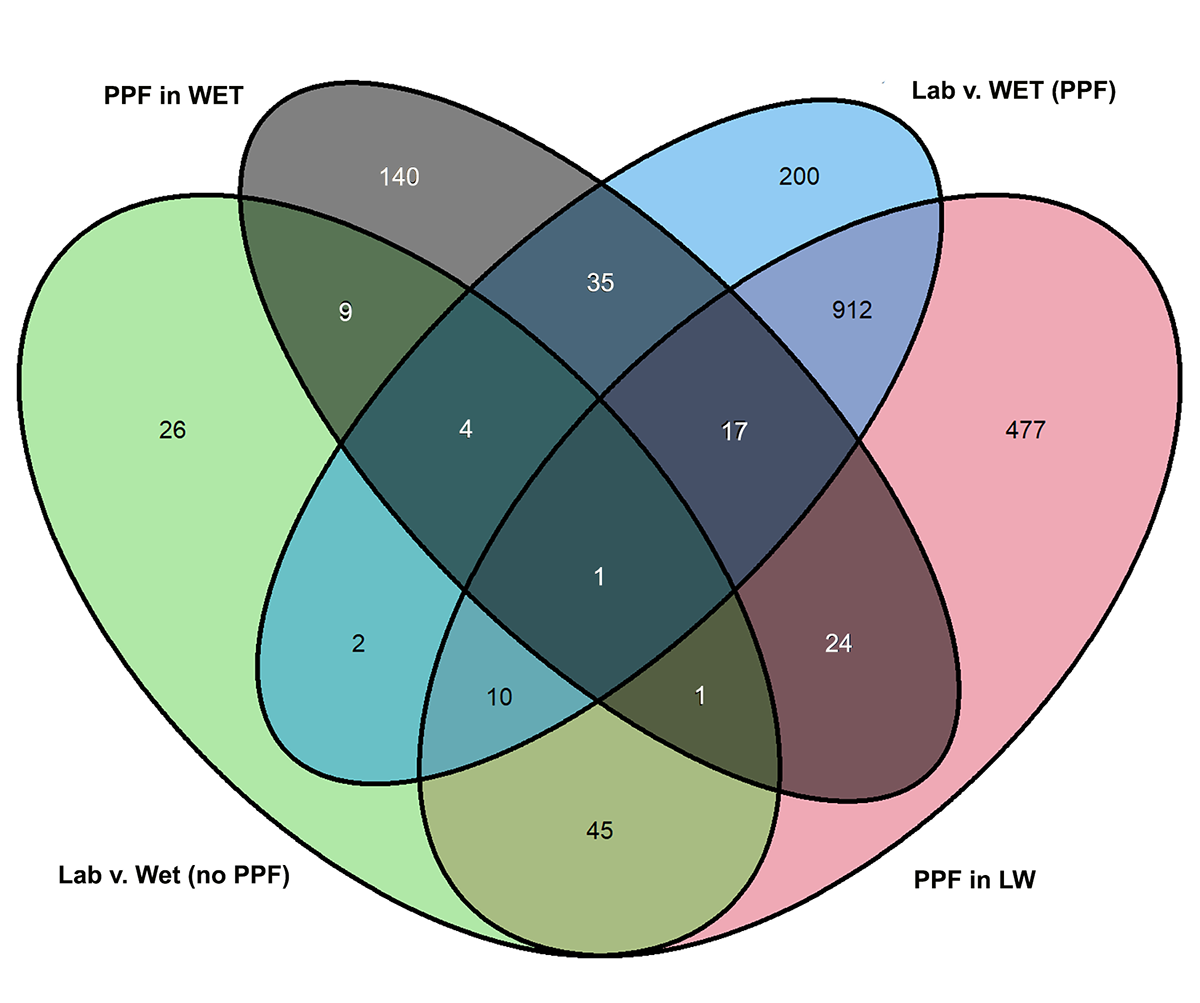

Supplement: Supplementary file 4 — Supporting information. [file ETC-41-2107-s007.tif]

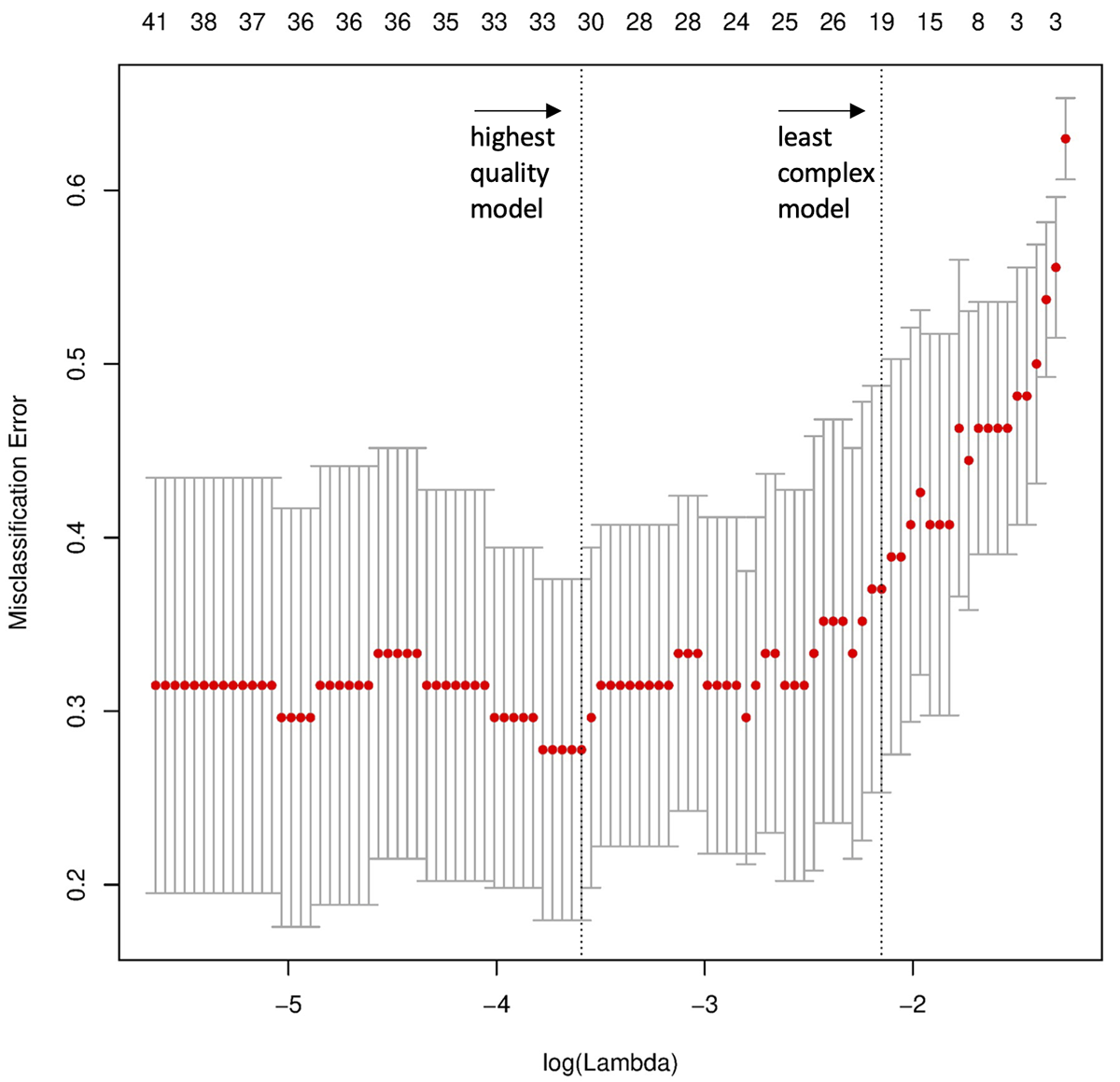

Supplement: Supplementary file 5 — Supporting information. [file ETC-41-2107-s003.tif]

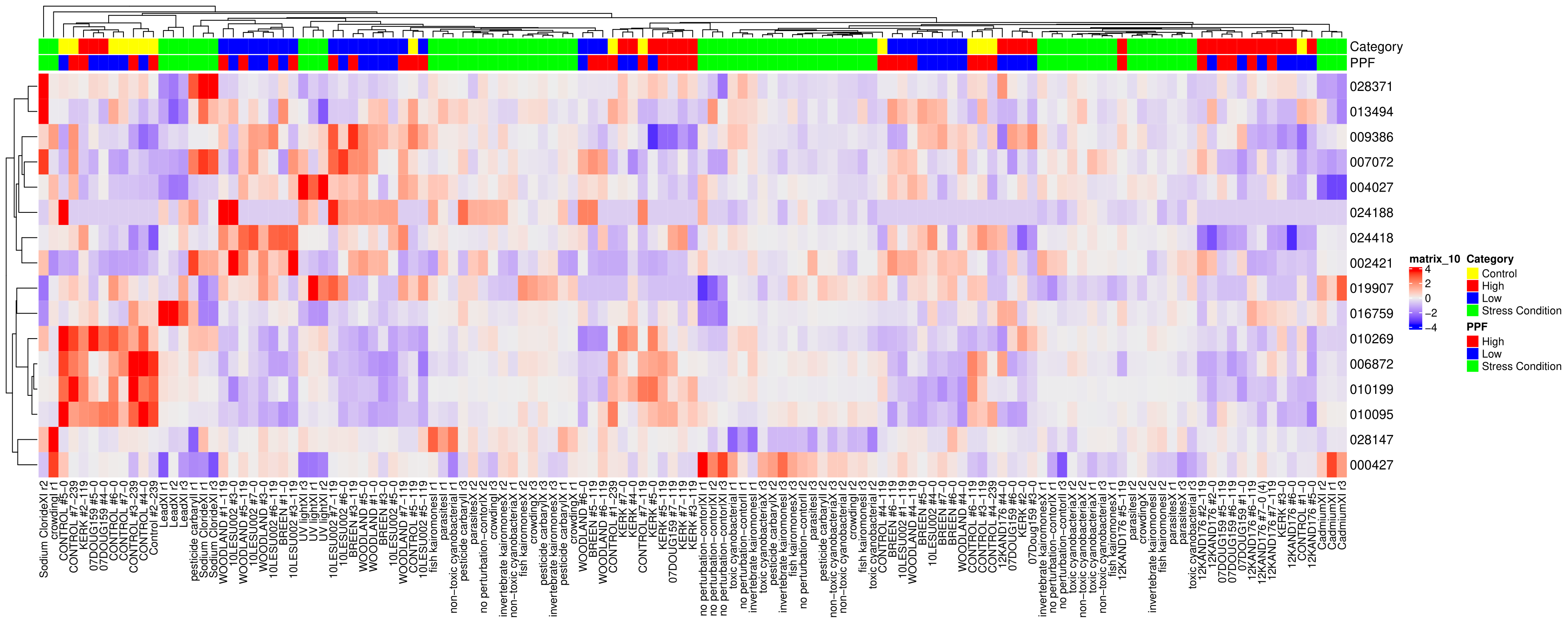

Supplement: Supplementary file 6 — Supporting information. [file ETC-41-2107-s006.tif]

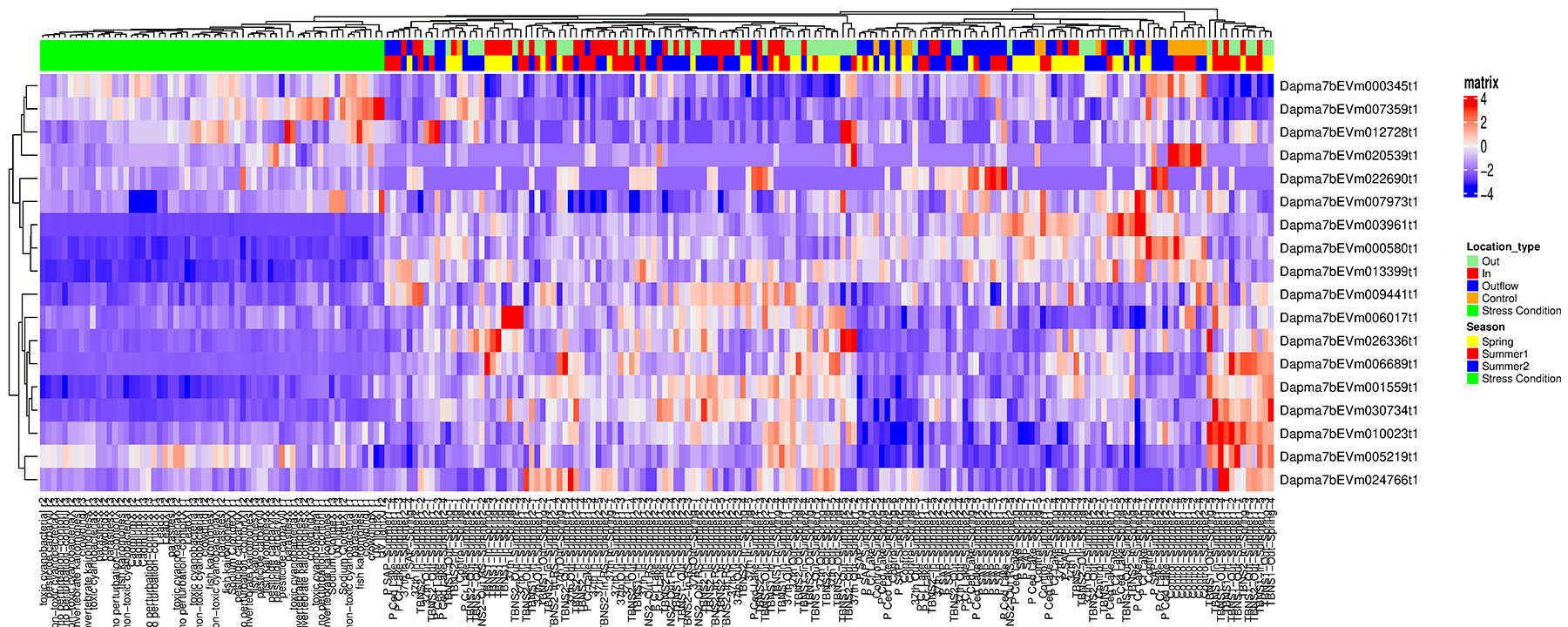

Supplement: Supplementary file 7 — Supporting information. [file ETC-41-2107-s002.tif]
